# Gene Transfer Potential of Outer Membrane Vesicles of Gram-Negative Bacteria

**DOI:** 10.3390/ijms22115985

**Published:** 2021-06-01

**Authors:** Federica Dell’Annunziata, Veronica Folliero, Rosa Giugliano, Anna De Filippis, Cristina Santarcangelo, Viviana Izzo, Maria Daglia, Massimiliano Galdiero, Carla Renata Arciola, Gianluigi Franci

**Affiliations:** 1 Department of Experimental Medicine, University of Campania Luigi Vanvitelli, 80138 Naples, Italy; federica.dellannunziata@unicampania.it (F.D.); veronica.folliero@unicampania.it (V.F.); rosa.giugliano@unicampania.it (R.G.); anna.defilippis@unicampania.it (A.D.F.); massimiliano.galdiero@unicampania.it (M.G.); 2 Department of Pharmacy, University of Naples Federico II, via Domenico Montesano 49, 80131 Naples, Italy; cristina.santarcangelo@unina.it (C.S.); maria.daglia@unina.it (M.D.); 3 Department of Medicine, Surgery and Dentistry Scuola Medica Salernitana, University of Salerno, 84081 Salerno, Italy; vizzo@unisa.it; 4 International Research Center for Food Nutrition and Safety, Jiangsu University, Zhenjiang 212013, China; 5 Research Unit on Implant Infections, Laboratorio di Patologia delle Infezioni Associate all’Impianto, IRCCS Istituto Ortopedico Rizzoli, 40136 Bologna, Italy; 6 Department of Experimental, Diagnostic and Specialty Medicine, University of Bologna, 40126 Bologna, Italy

**Keywords:** outer membrane vesicles, horizontal gene transfer, gram-negative bacteria, DNA

## Abstract

The increasing spread of multidrug-resistant pathogenic bacteria is one of the major threats to public health worldwide. Bacteria can acquire antibiotic resistance and virulence genes through horizontal gene transfer (HGT). A novel horizontal gene transfer mechanism mediated by outer membrane vesicles (OMVs) has been recently identified. OMVs are rounded nanostructures released during their growth by Gram-negative bacteria. Biologically active toxins and virulence factors are often entrapped within these vesicles that behave as molecular carriers. Recently, OMVs have been reported to contain DNA molecules, but little is known about the vesicle packaging, release, and transfer mechanisms. The present review highlights the role of OMVs in HGT processes in Gram-negative bacteria.

## 1. Introduction

The advent of multidrug-resistant (MDR) bacteria represents a serious global public health problem. Infections caused by MDR bacteria are second in the ranking of deadly diseases in developing countries [1]. According to the World Health Organization (WHO), approximately 700,000 people die from drug-resistant infections each year. In addition, 10 million deaths are predicted every year until 2050 [2,3,4]. Many successful bacterial infections are consequences of bacterial virulence mechanisms associated with antimicrobial escape [5]. Bacteria can escape antibiotic treatment and adapt to adverse environmental conditions through the development of different mechanisms of antibiotic resistance, while virulence mechanisms are essential to suppress and overcome the host’s defenses [6,7,8,9]. Several virulence factors and antibiotic-resistance genes can be transferred via horizontal gene transfer (HGT) [10]. Bacteria have developed several efficient and complex processes of HGT and currently, three are the most well-known mechanisms: (i) natural transformation; (ii) transduction; (iii) conjugation [11,12]. The natural transformation consists of the acquisition of free DNA from the environment, which is released from both living and dead cells [13]; the bacteria must be in a state of competence in order to acquire extracellular DNA. Natural competence is an event that is highly regulated, requiring more than 20 genes [14]. Approximately 1% of bacterial species are naturally transformable [15]. The transduction involves bacteriophages that transfer DNA to bacterial cells through infection. Bacteriophages can contain up to 100 kilobases of DNA, and the infection is limited to host specificity [16]. Conjugation requires cell-to-cell contact, mediated by the sexual pilus which connects donor cells with recipient cells. Plasmid transfer systems are strictly dependent on the characteristics of the genetic material to be transferred: conjugative plasmids contain genes necessary for pilus formation, while mobilizable plasmids carry mobility genes and transfer origin, lacking pilus coding genes [17,18]. Although these known mechanisms contribute to the gene flow within bacteria, they have restrictions such as limited genetic load, host specificity, and the kind of genetic material to be transferred. Recent studies have revealed a novel mechanism for gene transfer via OMVs. OMVs are spherical nanostructures (20–250 nm) naturally produced by Gram-negative bacteria [11,19,20]. These vesicles play crucial roles in bacterial virulence, modulation of host immune response, and intracellular communication [21,22,23]. Although these roles are widely studied, little is known about HGT mediated by OMVs. The main purpose of the present review is to analyse the available evidence for a thorough description of the implication of OMVs in the HGT mechanism.

## 2. Methodology

**Figure 1:**
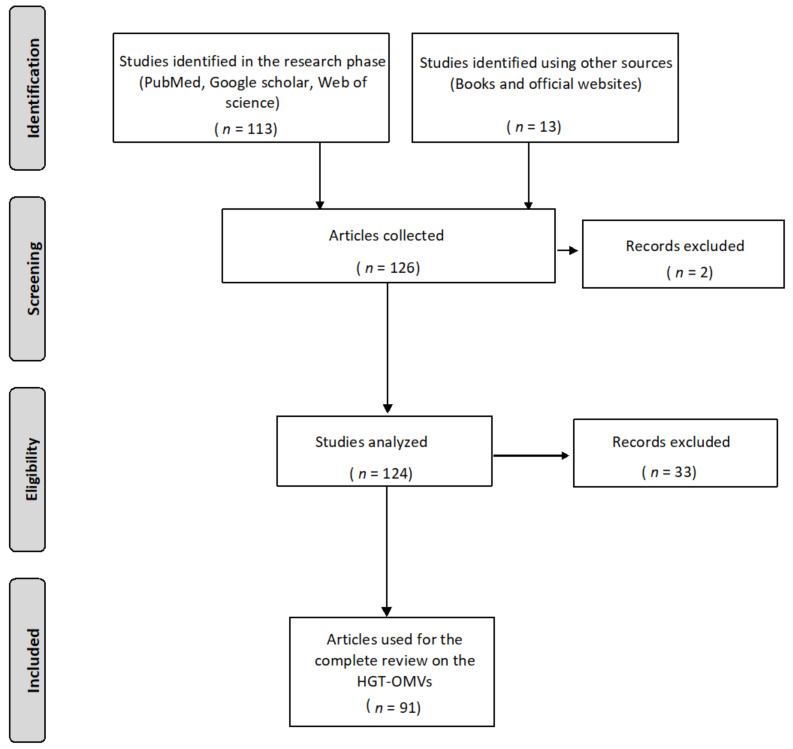
PRISMA flow diagram for the process of the study selection.

The present review consists of the first literature collection on the HGT mediated by OMVs secreted by Gram-negative bacteria. The purpose was to emphasize this novel gene-transfer method to better understand the mechanisms underlying the spread of antibiotic resistance. Various electronic databases were used for the literature search, including PubMed and Google scholar mainly, followed by Scopus and the Web of Science. For the complete article collection, the keywords used in the bibliographic research were “outer membrane vesicles”, “OMVs biogenesis”, “OMVs composition”, “OMVs roles”, “horizontal gene transfer”, “bacterial evolution”, transformation”, “transduction”, “conjugation”, “Gram-negative bacteria”, “antibiotic resistance”, and “resistance genes spreads”. The selection of articles was based on two main criteria: (i) more recent studies concerning the structure, composition, and function of OMVs; (ii) all experimental evidence regarding HGT-OMVs. Based on the chosen criteria, 126 articles published up to 2021 were found to be suitable. Of these articles, 91 were selected, summarized and critically discussed in order to provide a coherent review. Figure 1 illustrates the PRISMA flowchart for study selection. The following sections discuss all the evidence currently available regarding HGT-OMVs and their impact on gene resistance spread.

## 3. OMVs: An Overview from Structure to Function

### 3.1. Composition of the OMVs

**Figure 2:**
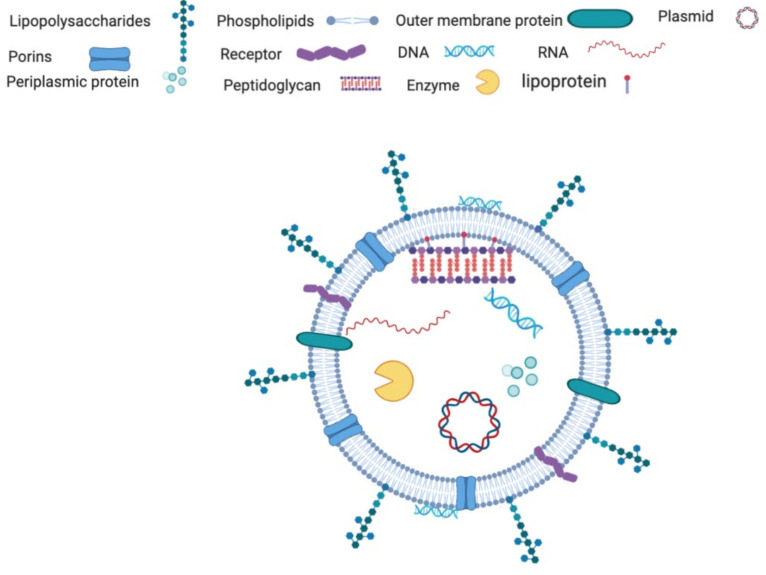
Typical composition of vesicles of OMV. Created with BioRender.com.

OMVs (Figure 2) are spherical bi-layered membrane nanostructures (50–500 nm), secreted by Gram-negative bacteria through bulging and ‘pinching off’ of the outer membrane [19]. Purification techniques have revealed the specific proteins and lipid composition of these vesicles [24]. The OMV membrane consists of lipopolysaccharide (LPS), phospholipids, membrane proteins, and peptidoglycan components, which mostly compose the structure of the outer membrane [25]. The lumen of the vesicles contains periplasmic proteins, cytosolic components, and nucleic acids [26]. The composition of OMVs is modulated by growth conditions and is also highly influenced from the interactions between host cells and bacteria [27,28].

#### 3.1.1. Protein Components

Several studies have demonstrated the presence within OMVs of outer membrane proteins, periplasmic proteins, and different virulence factors, engaged in the adhesion and invasion of cell hosts [29]. The advancement in proteomics techniques allowed the identification of more than 3500 proteins with different functions [30]. The identified proteins can be clustered in two main categories: (i) proteins of the outer membrane; and (ii) cargo proteins in the lumen. The first group mainly includes outer membrane proteins such as porins, components of transport systems, adhesins, phospholipases, and proteases. The porins widely identified in *Pseudomonas aeruginosa* OMVs were *OprF* and *OprH*/O*prG* [31]. In *Neisseria meningitidis* OMVs, porin A, factor H binding protein, and opacity-associated protein C represent the largest protein fraction [32]. Outer membrane phospholipase A was observed in the OMVs of *Shigella flexneri* [33]. OMVs from *Treponema denticola* contain active proteases that cause damage to host cells [34]. On the other hand, the second group mainly consists of toxins, such as the cholera toxin of *Vibrio cholerae*, cytolysin A of enterotoxic *Escherichia coli*, vacuolating cytotoxin of *Helicobacter pylori*, cytolethal distending toxin from *Campylobacter jejuni* [35], and enzymes such as proteases, glycosidases, and ureases [36].

#### 3.1.2. Lipid Components

Lipids play an essential role in the structure of OMVs and consist of phospholipids and lipopolysaccharides. While phospholipids constitute the inner sheet of the outer membrane, LPS is exclusively located on the outside surface of the outer membrane. Several studies have shown that the phospholipid content changes among various Gram-negative bacteria [37]. The phospholipid content of *Escherichia coli* OMVs consists mainly of phosphatidylethanolamine, phosphatidylglycerol, and lyso-phosphatidylethanolamine [38]. *N. meningitidis* OMVs mostly include phosphatidylglycerol and phosphatidylethanolamine [32]. In *P. aeruginosa,* the OMVs phosphatidylglycerol and stearic acid are abundantly detected, proving greater rigidity of the vesicles [39]. *Helicobacter pylori* OMVs have cardiolipin as the main lipid component [29]. The type of LPS band present on OMVs depends on the site of vesicle budding [19]. LPS possesses two distinct types of O polysaccharide, LPS of bands A and B. Their different chemical composition confers distinct surface and antigenic properties. Usually, A- and B-band LPS are present in the outer membrane [40]. In OMVs of *P. aeruginosa*, only B-band LPS are found, while in *Porphyromonas gingivalis,* OMVs are composed of A-band LPS [41].

#### 3.1.3. Nucleotide Component

OMVs carry DNA and RNA on their surface or in the vesicular lumen. A clear difference can be observed by OMV treatment with DNase and RNase: luminal DNA and RNA are preserved even after the enzymatic treatment [40,42]. Several studies showed the presence of DNA in the OMV lumen of *Escherichia coli* [43], *Neisseria gonorrhoeae* [44], *P. aeruginosa* [45], *Acinetobacter baumannii* [46], etc. Small RNA sequences were detected in *Vibrio cholerae* and *P. aeruginosa* OMVs [47,48]. Koeppen et al. demonstrated that OMV-associated small RNA contributes to the pathogenicity of *P. aeruginosa* by reducing the host’s immune response [48]. Furuse et al. have recently identified tRNA fragments in the OMVs of *Chlamydia* and *Legionella* strains, involved in direct subversion of host gene translation and mRNA stability [49]. Although novel information on this topic is piling up at a steady pace, nucleic acid incorporation mechanisms remain to be elucidated.

#### 3.1.4. Biogenesis 

OMV biogenesis occurs through a budding process from the outer membrane [19]. Three different models for OMV production have been proposed based on several biochemical and genetical studies [50]. In the first model, Burdett et al. suggested that the absence or transfer of covalent links between the outer membrane and peptidoglycan layer promotes vesiculation [51]. Mutations in Braun lipoprotein [52], the Tol-Pal system of *Escherichia coli* [53], and RmpM of *N. meningitidis* [54] demonstrated the importance of the outer membrane peptidoglycan-linking structures to increase OMV production. The second model showed that the accumulation of peptidoglycan fragments or poorly folded proteins in periplasmic space exert pressure on the outer membrane, determining the curvature of the membrane and final budding [25]. In the third model, Mashburn et al. identified molecules that induce the curvature of the membrane such as the *Pseudomonas* quinolone signal (PQS) and the B-band lipopolysaccharide [55,56]. PQS acts not only as a vesiculation-inducing signal but also as a direct effector of OMV formation [57]. Mashburn et al. demonstrated that PSQ induces membrane curvature, binding phospholipids and lipopolysaccharides [58]. Sequestration of positively charged compounds (Mg^2+^ and Ca^2+^ salt) by PQS causes anionic repulsion of lipopolysaccharides, increasing vesiculations [59]. Further, the presence of B-band lipopolysaccharide in *P. aeruginosa* contributes to the curvature of the membrane, inducing the repulsion of lipopolysaccharides [60]. Berry et al. demonstrated that a cell-wall mutant of *P. aeruginosa*, unable to synthesize B-band LPS, exhibited impaired OMV production [61]. The biogenesis of vesicles begins during the exponential growth phase, reaching a maximum peak in the stationary growth phase [29,40]. However, several factors (temperature, culture medium, presence of antibiotics) qualitatively and quantitatively influence the production of OMVs. Baumgarten et al. showed that the production of *Pseudomonas putida* OMVs under thermal stress conditions (grown at 55 °C) was substantially increased [39]. In the study by Bauwens et al., the vesiculation was increased in response to nutrient limitation and exposure to chemical or physical stress-inducing factors; this increase in vesiculation represents an adaptation mechanism to the host environment during infection [62]. These studies indicate that bacteria developed the OMV production mechanism as a part of stress response to ensure bacterial survival [63].

### 3.2. Biological Functions

Several biological functions are attributed to the OMVs. These vesicles represent a long-distance delivery system of biomolecules, such as nucleic acids, enzymes, toxins, and virulence factors, protecting them from extracellular degradation and dilution. 

Vesiculation allows intraspecies and interspecies communications and contributes to interaction with the host [21]. In addition, OMVs are involved in the acquisition of nutrients, stress responses, and the formation of a microenvironment necessary for the survival of pathogens [28,64,65]. The packaging of proteases, phosphatase, and glycosidases in OMVs plays an important role in the degradation of complex molecules, promoting nutrient availability [36]. Evans et al. have shown that alkaline phosphatase in *Myxococcus xanthus* OMVs causes the release of phosphate, contributing to the development of multicellular communities [66]. In addition, proteins and DNA associated with the OMV surface represent a source of carbon and nitrogen during bacterial growth [53].

OMVs participate in the formation of the biofilm matrix by the release of exopolysaccharides, thus increasing cell co-aggregation [55,67]. *P. aeruginosa* is able to form biofilms and cause surgical site infections, orthopedic peri-implant bone infections, and lung infection in cystic fibrosis patients. Cooke et al. showed that the *Pseudomonas* quinolone signal (PQS) induces OMV formation in *P. aeruginosa* and in other species of the *Pseudomonas* genus through a biophysical mechanism that is highly dynamic during biofilm development. Noticeably, OMV synthesis is significantly elevated during dispersion, compared to attachment and maturation stages, so the authors suggested that PQS-induced OMVs enhance biofilm dispersion in *P. aeruginosa* infections, thus promoting biofilm dissemination [68]. Seike et al. purified OMVs from several *Aeromonas* species and examined their effect on biofilm formation. They found that the addition of OMVs promoted biofilm formation in a dose-dependent manner (except for one strain, which turned out to be non-producer). These results suggest that the OMVs released from the bacterial cells are closely related to the biofilm formation of the *Aeromonas* genus [69]. OMVs play important roles in microbial virulence and in modulating the host immune response [28]. PQS biosynthetic and receptor mutant biofilms were significantly impaired in their ability to disperse, but this phenotype could be rescued by genetic complementation or exogenous addition of PQS. Finally, OMVs participate in extracellular protein, lipid, and nucleotide degradation. Thus, PQS-induced vesiculation could play an essential role in the degradation of biofim matrix components, facilitating cell escape. Toledo et al. reported that phosphoenolpyruvate, present in the vesicular lumen, induced biofilm degradation, contributing to the colonization of the host by the bacterium [70]. Baarda et al. showed that *N. gonorrhoeae* OMVs sequester antibodies in the surrounding environment, protecting bacteria from the opsonization process [71]. The bacterium–host interactions trigger the release of OMVs, which carry toxins and adhesion and virulence factors [72]. Kuehn et al. showed that the vesicles implement the adhesion of bacteria to the surface of the host cells, acting as a bridge [73]. Bielaszewska et al. demonstrated that OMVs increase the adhesion of bacteria to the intestinal epithelium, aiding them to resist physical elimination [74]. Toxins and virulence factors carried by OMVs are more active than their soluble forms [25]. The Shiga toxin in *Escherichia coli* OMVs efficiently inhibits eukaryotic protein synthesis compared to the soluble form [62]. OMVs contain several pathogenic molecular patterns, including porins and lipopolysaccharides [75]. These strongly modulate the immune response, leading the production of cytokines and chemokines, which induce the activation of the inflammatory response [76,77].

In addition to the reported functions, OMVs have recently been recognized as gene transfer vectors [78]. Several studies have detected plasmids, chromosomal DNA fragments, bacteriophage DNA and RNA fragments in OMVs [22,79,80,81]. Therefore, in the following section, recent evidence on the role of OMVs as carriers for horizontal gene transfer will be reported. 

## 4. Horizontal Gene Transfer Mediated by OMVs

**Figure 3:**
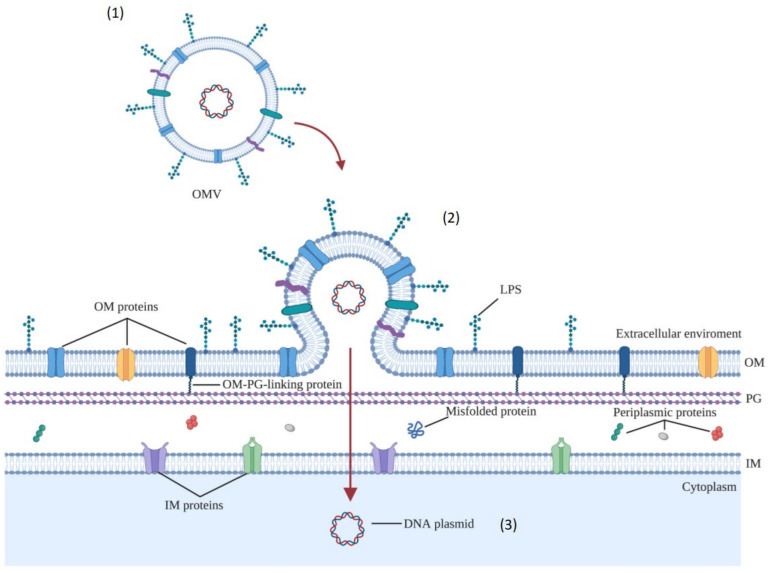
Horizontal gene transfer mediated by OMVs. (**1**) DNA is released through OMVs from donor cells in the surrounding environment; (**2**) OMVs merge with the outer membrane of the recipient cell; and (**3**) genetic material migrates into the cytoplasm giving the bacterium new adaptive capacities through the expression of the genetic material acquired [19]. Created with BioRender.com.

Gene transfer can occur via proven processes of transformation, conjugation, and transduction, as well as through recently identified OMV-mediated mechanisms (Figure 3) [13]. Few studies have evaluated the gene transfer potential of OMVs (Table 1).

Kolling et al. were the first to identify OMVs as gene transfer vectors. In this study, OMVs from *Escherichia coli* O157:H7 were purified, and the DNA content was evaluated. The vesicles were treated with DNase to demonstrate the intravesicular gene localization. Polymerase chain reaction (PCR) data revealed the presence of the *eae*, *uidA*, *stx1,* and *stx2* virulence genes in the luminal space. These genes were transferred to *Escherichia coli* JM109 through OMV-recipient cell contact. HGT was proved through PCR amplification of virulence genes in transformed *Escherichia coli* JM109 [43]. These first findings laid the foundations for other investigations, deepening the role of OMVs in gene transfer mechanisms.

Yaron et al. proved that genetic exchanges through OMVs can also occur between bacteria of different species. In this study, OMVs were isolated from a culture supernatant of *Escherichia coli* O157:H7. DNase treatment and PCR analysis allowed the intravesicular detection of the chromosomal genes *eaeA* and *uidA*, genes *stx1* and *stx2* of bacteriophage origin, and of *hlyCA*, *L7095*, and *mobA*, associated with pO157, pO157, and p4821 plasmids, respectively. Transformation experiments were performed, using *Escherichia coli* JM109 and *Salmonella enterica* serovar Enteritidis as recipient cells, and target genes were determined by colony PCR amplification. The acquisition of virulence genes in the recipient cells resulted in an increase in pathogenicity; the latter was assessed by Vero cell assay. Vero cells were treated with transformed and untransformed bacteria, and the cytotoxic effect was evaluated. The transformed recipient strains induced a cytotoxicity six times higher than the unprocessed strains, indicating the expression of virulence factors only in transformed strains. This study proved a transfer independent of the phylogenetic correlation between donor and recipient cells [82].

Besides *E. coli*, other Gram-negative species exploit OMVs as HGT vectors. Ho et al. proved that *P. gingivalis* OMVs mediated the transfer of virulence genes between members of the same species. Genes encoding the major subunit of long fimbriae (*fimA*) and superoxide dismutase (*sod*) were detected in the vesicular lumen by PCR analysis, suggesting possible preferential DNA packaging. OMV–HGT experiments were conducted using a mutant *P. gingivalis* 49,417, obtained by introducing a 2.1 Kb segment of the *ermF-ermAM* gene into the *fimA* gene, which conferred resistance to erythromycin. The produced OMVs transferred both virulence and erythromycin resistance genes to sensitive strains of *P. gingivalis* 33,277. These findings indicated the OMVs act as an offensive arm of *P. gingivalis* in the host oral cavity [84].

The involvement of OMVs in the spread of resistance genes was only revealed 10 years later. Rumbo et al. identified for the first time OMVs as vectors of antibiotic resistance gene transfer. In this study, two carbapenem-resistant clinical strains of *A. baumannii*, which carry the plasmid-borne *bla*_OXA-24_ gene, were used to purify OMVs. DNase treatment and dot-blot experiments demonstrated the presence of the *bla*_OXA-24_ gene within OMVs from carbapenem-resistant clinical strains. Vesicular contact with carbapenem-sensitive *A. baumannii* induced total resistance to carbapenems. PCR amplification of *bla*_OXA-24_ gene and minimal inhibitory concentration (MIC) values of imipenem, meropenem, and doripenem for the transformed strain were estimated. The presence of genes and increased MIC value (>32 μg) confirmed the acquisition of resistance. Time–response experiments revealed that the transformation occurs within 3 h of incubation and increases to 24 h, reaching a plateau [85]. Chatterjee et al. also supported the transfer of antibiotic resistance genes through OMVs. They considered the transmission of the New Delhi metallo-β-lactamase-1 (*bla*_NDM-1_) gene via lipidic vesicles released from *A. baumannii* strain A_115. DNase treatment, PCR, and dot-blot analysis proved the presence of the *bla*_NDM-1_ gene in the vesicular lumen. The transformation of β-lactam sensitive strains of *A. baumannii* and *Escherichia coli* JM109 was conducted with different amounts of OMVs. The number of transformants grew at increasing OMVs amounts, added to the receiving cells. The highest transformation efficiency (4.62 × 10^9^ CFU/mL) was recorded following the treatment of *A. baumannii* ATCC 19606 with 50 µg of OMVs. No transformants were attained when free plasmid was incubated with β-lactam sensible strains, proving that the transfer was mediated exclusively by the vesicle. The recipient cells were positive for the *bla*_NDM-1_ gene and exhibited a broad profile of resistance to β-lactam antibiotics, recording higher MIC values, compared to untransformed strains. Moreover, the EcoRI plasmid digestion profile of plasmids isolated from transformed strains overlapped with the one isolated from *A. baumannii* A_115. This study demonstrated the intra-species and inter-species transfer of a whole plasmid via OMVs [46].

DNA packaging into vesicular lumens protected it from adverse environmental conditions, demonstrating an additional bacterial survival advantage associated with OMV–HGT. For instance, the vesicles ensure gene transfer in thermal environments. Under these conditions, the integrity of the extracellular DNA is compromised by the high temperatures and the action of DNase. In *Thermus thermophilus*, the mechanisms of transformation and conjugation mainly participate in HGT. Blesa et al. detected another transformation pathway based on OMVs. The vesicles from *Thermus thermophilus*, transformed with the pMKpnqosYFP plasmid, were purified. After DNase treatment of OMVs, plasmid integrity in the vesicles was evaluated through agarose gel electrophoresis and HindIII plasmid digestion. Vesicular plasmid integrity was not compromised, which indicates that OMVs provide protection for the DNA within them. Transformation experiments were conducted using *ΔpilQ* and *ΔpilA4 Thermus thermophilus* as recipient strains. In the thermal environments and in the presence of DNase, OMVs increased the frequency of transformation, compared to free DNA. These findings support the role of OMVs as vehicles for HGT under adverse conditions [86].

Despite different studies that have shown a high gene transfer potential of OMVs, Renelli et al. suggested that vesicles carry the genetic material but do not promote an effective transformation. OMVs produced by *P. aeruginosa* PAO1, transformed with the pAK1900 plasmid (p-OMVs), were isolated and analyzed for their DNA content. DNase treatment and fluorometric analysis detected DNA in the vesicular lumen of p-OMV. PCR amplification of plasmid (pAK1900) and chromosomal (*oprL*) sequences indicated the presence of only plasmid DNA within vesicles. The transformation experiments were performed using *P. aeruginosa* PAO1 and *Escherichia coli* DH5a as recipient strains. PCR results showed that p-OMVs were unable to transform the PAO1 and DH5a strains. The author speculated that p-OMVs transferred the plasmid into the periplasm of recipient cells, which does not by-pass the plasma membrane for efficient transformation [87].

### Factors Affecting Horizontal Gene Transfer Mediated by OMV

Although previous studies have highlighted the role of OMVs in HGT, the mechanisms underlying the transfer and the factors influencing the process are not yet clear [79,89]. Few studies have been conducted to understand these aspects. 

Tran et al. evaluated the role of plasmid type and recipient/donor strains on gene transfer rates. In this research, *Escherichia coli* strains were transformed with pLC291, pUC19, and pZS2501 plasmids, and OMVs were purified. The selected plasmids had similar size but different replication origin. pLC291 and pUC19 are high-copy number plasmids, while pZS2501 is a low-copy plasmid. The three plasmids were proved to be present in *Escherichia coli* OMVs through PCR analysis. The protein content of the three vesicles was similar, but only vesicles containing pZS2501 showed an increased size. The incorporation rate of plasmid in the OMVs was evaluated by real time PCR analysis. The low-copy number plasmid had a low loading capacity; in particular, 0.49 × 10^3^ copies per pg of vesicle protein were detected. The high-copy number pLC291 and pUC19 plasmids showed a high loading potential with 2.58 × 10^3^ and 482.7 × 10^3^ plasmid copies per pg of OMVs protein, respectively. These findings showed that plasmid features influenced vesicle diameter and plasmid loading. Furthermore, they investigated whether the OMVs released by different recipient strains were endowed with different characteristics. In a transformation experiment, *Aeromonas veronii*, *Enterobacter cloacae,* and *Escherichia coli* were used as recipient strains. *Escherichia coli*, transformed with the pLC291 plasmid, was used as the donor strain. Purified vesicles from different recipient strains contained the same protein and plasmid amounts and had a similar size. In addition, they evaluated the potential of OMVs to perform interspecies gene transfer. The vesicles containing pLC291, isolated from *Aeromonas veronii*, *Enterobacter cloacae,* and *Escherichia coli*, were exploited to induce the transformation of five different recipient strains, i.e., *Aeromonas veronii*, *Enterobacter cloacae*, *Escherichia coli*, *Chromobacterium violaceum,* and *P. aeruginosa*. The time to plasmid transfer was quantified for each donor/recipient strain. *Aeromonas veronii* transferred pLC291 via OMVs to different recipient strains in less time. *P. aeruginosa* acquired antibiotic resistance in a shorter time, regardless of the donor species. *Aeromonas veronii* received the plasmid faster than *Enterobacter cloacae*, *Escherichia coli*, and *Chromobacterium violaceum*. These results showed that the ability to acquire DNA may depend on the species of the donor/recipient bacteria [88].

In a subsequent investigation, Tran et al. evaluated more closely the effect of plasmid features, such as plasmid copy number, size, and origin of replication on OMV-mediated gene transfer. Three specific point mutations in the pSC101 plasmid origin of the replication were introduced to increase the number of copies. pSC101^+^, pSC101^++^, and pSC101^+++^ with increasing numbers of copies were electroporated into the donor *Escherichia coli* strains. Increased plasmid copy number did not affect the size and the amount of OMVs purified but changed the number of plasmids loaded into vesicles. Transfer experiments showed that the plasmid transfer time decreased by increasing the plasmid copy number. In addition, this study assessed the impact of plasmid size on vesicle loading. Four plasmids of different sizes, based on the plasmid pLC291, were generated through the insertion of non-functional lambda phage DNA. The obtained plasmids had dimensions of 3.5, 7, 10, and 15 kb, named pLC-3.5, pLC-7, pLC10, and pLC15, respectively. Strains of *Escherichia coli* were transformed with each plasmid, and OMVs were purified. OMV production scarcely increased as the plasmid dimensions increased. Dynamic light scattering analysis showed that vesicle dimension was independent of the plasmid size. Moreover, qPCR results showed that the plasmid size inversely affected the number of plasmid copies in the vesicle. To assess the impact of plasmid origin on OMV production, they constructed three plasmids based on plasmid pLC291, with the same size (3.5 kb) but different origins: pMB1, pLC with dual origins of RK2 and ColE1, and SC101. The vesicles purified from *Escherichia coli* transformed with each plasmid were analyzed. The production and size of the vesicles were similar among OMVs with different origins. The packaging was different for each type of plasmid (pMB1, pLC, and SC101): 364.45 × 10^3^ copies per pg of vesicle protein for the plasmid origin pMB1, while 3.13 × 10^3^ and 1.12 × 10^3^ copies per pg of vesicle protein for pLC and SC101 were observed, respectively. OMV-mediated gene transfer experiments were conducted, using *Escherichia coli* as recipient strains, and the same amounts of OMVs were added. The gene transfer rate was strongly influenced by the plasmid origin of replication. In particular, vesicles including pLC plasmids had a transfer rate roughly 10 times higher than the vesicles containing pMB1 and SC101 plasmids. Together, these findings showed that the number and size of the plasmid influenced the packaging efficiency into vesicles, while the origin of the replication affected the absorption rate of the vesicles [90].

Only one study evaluated the impact of external factors on vesicle packaging. Fulsundar et al. purified OMVs from *Acinetobacter baylyi* transformed with the pMU125 plasmid in the presence of stressors such as high temperature, desiccation, nutrient deprivation, UV light, and antibiotic exposure (gentamicin and chloramphenicol). The vesicles purified by treated bacteria were characterized on the basis of protein and DNA contents. High temperature, desiccation, nutrient deprivation, UV light, and antibiotic exposure caused the increased release of vesicles. A significant rise in the amount of plasmid in OMVs was observed when the bacteria were treated with chloramphenicol and gentamicin and were grown in the absence of nutrients. The authors suggested that this could increase the frequency of plasmid transfer. OMVs released from bacterial populations subjected to stress varied in size and zeta potential. Treatment of bacteria with antibiotics and nutrient deprivation caused a significant increase in vesicular diameters. The zeta potential of the OMV produced in the presence of gentamicin showed more negative values compared to the other treatments. These results proved that stress factors can influence the vesicle release, DNA content, and vesicle size [83].

## 5. Concluding Remarks

HGT is responsible for the exchange of genetic elements between bacterial cells and plays an important role in the evolution of many microorganisms [79]. The three known mechanisms of gene transfer, natural transformation, transduction, and conjugation, contribute significantly to the genetic diversity among bacterial species [91]. Features of genetic material and donor/recipient strain specificity are known to restrict gene transfer. Given these limitations, other pathways were thought to contribute to the gene exchange. Recently, an alternative mechanism of HGT via OMVs was identified. Several studies showed that OMVs carry DNA in their lumen, defining a further mechanism of gene dissemination, encoding virulence and antibiotic resistance factors. The cited studies underline the importance of OMVs not only for virulence in the processes of infection or host interaction, but also in the diffusion of genetic elements to surrounding bacteria [43,46,82,84,85,86,89] and in promoting biofilm formation and dissemination, which are prominent pathogenetic mechanisms of chronic infections, especially those associated with orthopedic implants. Although several studies have shown the occurrence of OMV-mediated HGT, little evidence has defined the mechanism and the factors that intervene in gene delivery [88]. Recent evidence showed that the identity of the genetic element and donor/recipient strains affect gene transfer rates. Stress factors such as high temperatures, nutrient deprivation, UV, and antibiotic treatments have also been seen to influence transfer efficiency [83]. These data suggest that OMVs contribute to gene transfer events. However, further studies are needed to better understand the vesicular loading mechanism, the absorption by the receiving strain, and the factors involved in the process.

## Figures and Tables

**Table 1:** Experimental studies of horizontal gene transfer mediated by OMVs.

Donor Bacterial Species	Genetic Material Transferred	Recipient Bacterial Cells	References
*Escherichia coli* O157: H7	*stx1 stx2* and *eae* and *uidA*	*Escherichia coli* JM109	[43]
*Escherichia coli* O157: H7	*eaeA*, *stx1*, *stx2*, *hlyCA*, *L7095,* and *mobA*	*Escherichia coli* JM109, *Salmonella enteritidis*	[82]
*Acinetobacter baylyi*	plasmid pMU125	*Escherichia coli*, *Acinetobacter baylyi*	[83]
*Porphyromonas gingivalis*	*fimA* and *sod*	*Porphyromonas. gingivalis*	[84]
*Acinetobacter baumannii*	*bla*_OXA-24_	*Acinetobacter baumannii*	[85]
*Acinetobacter baumannii*	*bla*_NDM-1_	*Acinetobacter baumannii,* *Escherichia coli*	[46]
*Thermus thermophilus*	plasmid pMKpnqosYFP	*Thermus thermophilus*	[86]
*Pseudomonas aeruginosa*	plasmid pAK1900	*Pseudomonas aeruginosa*, *Escherichia coli*	[87]
*Escherichia coli*	plasmids pLC291, pUC19, and pZS2501	*Aeromonas veronii*, *Enterobacter cloacae, Escherichia coli*	[88]

## References

[1] Abadi A.T.B., Rizvanov A.A., Haertlé T., Blatt N.L. (2019). World Health Organization Report: Current crisis of antibiotic resistance. BioNanoScience.

[2] Sugden R., Kelly R., Davies S. (2016). Combatting antimicrobial resistance globally. Nat. Microbiol..

[3] European Food Safety Authority, European Centre for Disease Prevention and Control (2018). The European Union summary report on antimicrobial resistance in zoonotic and indicator bacteria from humans, animals and food in 2016. EFSA J..

[4] WHO National Action Plans and Monitoring and Evaluation, Surveillance, Prevention and Control. https://ahpsr.who.int/publications/i/item/global-action-plan-on-antimicrobial-resistance.

[5] Beceiro A., Tomas M., Bou G. (2013). Antimicrobial resistance and virulence: A successful or deleterious association in the bacterial world?. Clin. Microbiol. Rev..

[6] Kitamoto S., Nagao-Kitamoto H., Kuffa P., Kamada N. (2016). Regulation of virulence: The rise and fall of gastrointestinal pathogens. J. Gastroenterol..

[7] Li B., Webster T.J. (2018). Bacteria antibiotic resistance: New challenges and opportunities for implant-associated orthopedic infections. J. Orthop. Res..

[8] Petrillo F., Pignataro D., Lavano M.A., Santella B., Folliero V., Zannella C., Astarita C., Gagliano C., Franci G., Avitabile T. (2020). Current evidence on the ocular surface microbiota and related diseases. Microorganisms.

[9] Blair J.M.A., Webber M.A., Baylay A.J., Ogbolu D.O., Piddock L.J.V. (2015). Molecular mechanisms of antibiotic resistance. Nat. Rev. Genet..

[10] Deng Y., Xu H., Su Y., Liu S., Xu L., Guo Z., Wu J., Cheng C., Feng J. (2019). Horizontal gene transfer contributes to virulence and antibiotic resistance of Vibrio harveyi 345 based on complete genome sequence analysis. BMC Genom..

[11] Soucy S.M., Huang J., Gogarten J.P. (2015). Horizontal gene transfer: Building the web of life. Nat. Rev. Genet..

[12] Gilbert C., Pace J.K., Feschotte C. (2009). Horizontal SPINning of transposons. Commun. Integr. Biol..

[13] Johnston C., Martin B., Fichant G., Polard P., Claverys J.P. (2014). Bacterial transformation: Distribution, shared mechanisms and divergent control. Nat. Rev. Microbiol..

[14] Mell J.C., Redfield R.J. (2014). Natural competence and the evolution of DNA uptake specificity. J. Bacteriol..

[15] Nazarian P., Tran F., Boedicker J.Q. (2018). Modeling Multispecies Gene Flow Dynamics Reveals the Unique Roles of Different Horizontal Gene Transfer Mechanisms. Front. Microbiol..

[16] Chiang Y.N., Penades J.R., Chen J. (2019). Genetic transduction by phages and chromosomal islands: The new and noncanonical. PLoS Pathog..

[17] Cabezon E., Ripoll-Rozada J., Pena A., de la Cruz F., Arechaga I. (2015). Towards an integrated model of bacterial conjugation. FEMS Microbiol. Rev..

[18] Fang Z., Zhou H. (2020). Identification of the conjugative and mobilizable plasmid fragments in the plasmidome using sequence signatures. Microb. Genom..

[19] Kulp A., Kuehn M.J. (2010). Biological functions and biogenesis of secreted bacterial outer membrane vesicles. Annu. Rev. Microbiol..

[20] Martora F., Pinto F., Folliero V., Cammarota M., Dell’Annunziata F., Squillaci G., Galdiero M., Morana A., Schiraldi C., Giovane A. (2019). Isolation, characterization and analysis of pro-inflammatory potential of Klebsiella pneumoniae outer membrane vesicles. Microb. Pathog..

[21] Berleman J., Auer M. (2013). The role of bacterial outer membrane vesicles for intra- and interspecies delivery. Environ. Microbiol..

[22] Perez-Cruz C., Carrion O., Delgado L., Martinez G., Lopez-Iglesias C., Mercade E. (2013). New type of outer membrane vesicle produced by the Gram-negative bacterium Shewanella vesiculosa M7T: Implications for DNA content. Appl. Environ. Microbiol..

[23] Bonnington K.E., Kuehn M.J. (2014). Protein selection and export via outer membrane vesicles. Biochim. Biophys. Acta.

[24] Klimentova J., Stulik J. (2015). Methods of isolation and purification of outer membrane vesicles from gram-negative bacteria. Microbiol. Res..

[25] Schwechheimer C., Kuehn M.J. (2015). Outer-membrane vesicles from Gram-negative bacteria: Biogenesis and functions. Nat. Rev. Microbiol..

[26] Qing G., Gong N., Chen X., Chen J., Zhang H., Wang Y., Wang R., Zhang S., Zhang Z., Zhao X. (2019). Natural and engineered bacterial outer membrane vesicles. Biophys. Rep..

[27] Orench-Rivera N., Kuehn M.J. (2016). Environmentally controlled bacterial vesicle-mediated export. Cell Microbiol..

[28] Cecil J.D., Sirisaengtaksin N., O’Brien-Simpson N.M., Krachler A.M. (2019). Outer membrane vesicle-host cell interactions. Microbiol. Spectr..

[29] Yu Y.J., Wang X.H., Fan G.C. (2018). Versatile effects of bacterium-released membrane vesicles on mammalian cells and infectious/inflammatory diseases. Acta Pharm. Sin..

[30] Kim Y.S., Choi E.J., Lee W.H., Choi S.J., Roh T.Y., Park J., Jee Y.K., Zhu Z., Koh Y.Y., Gho Y.S. (2013). Extracellular vesicles, especially derived from Gram-negative bacteria, in indoor dust induce neutrophilic pulmonary inflammation associated with both Th1 and Th17 cell responses. Clin. Exp. Allergy.

[31] Chevalier S., Bouffartigues E., Bodilis J., Maillot O., Lesouhaitier O., Feuilloley M.G.J., Orange N., Dufour A., Cornelis P. (2017). Structure, function and regulation of Pseudomonas aeruginosa porins. FEMS Microbiol. Rev..

[32] Gerritzen M.J.H., Stangowez L., van de Waterbeemd B., Martens D.E., Wijffels R.H., Stork M. (2019). Continuous production of Neisseria meningitidis outer membrane vesicles. Appl. Microbiol. Biotechnol..

[33] Wang X., Jiang F., Zheng J., Chen L., Dong J., Sun L., Zhu Y., Liu B., Yang J., Yang G. (2016). The outer membrane phospholipase A is essential for membrane integrity and type III secretion in Shigella flexneri. Open Biol..

[34] Chi B., Qi M., Kuramitsu H.K. (2003). Role of dentilisin in Treponema denticola epithelial cell layer penetration. Res. Microbiol..

[35] Van der Pol L., Stork M., van der Ley P. (2015). Outer membrane vesicles as platform vaccine technology. Biotechnol. J..

[36] Valguarnera E., Scott N.E., Azimzadeh P., Feldman M.F. (2018). Surface exposure and packing of lipoproteins into outer membrane vesicles are coupled processes in bacteroides. mSphere.

[37] Kohl P., Zingl F.G., Eichmann T.O., Schild S. (2018). Isolation of outer membrane vesicles including their quantitative and qualitative analyses. Methods Mol. Biol..

[38] Tashiro Y., Inagaki A., Shimizu M., Ichikawa S., Takaya N., Nakajima-Kambe T., Uchiyama H., Nomura N. (2011). Characterization of phospholipids in membrane vesicles derived from Pseudomonas aeruginosa. Biosci. Biotechnol. Biochem..

[39] Baumgarten T., Sperling S., Seifert J., von Bergen M., Steiniger F., Wick L.Y., Heipieper H.J. (2012). Membrane vesicle formation as a multiple-stress response mechanism enhances Pseudomonas putida DOT-T1E cell surface hydrophobicity and biofilm formation. Appl. Environ. Microbiol..

[40] Jan A.T. (2017). Outer Membrane Vesicles (OMVs) of gram-negative bacteria: A perspective update. Front. Microbiol..

[41] Veith P.D., Chen Y.Y., Gorasia D.G., Chen D., Glew M.D., O’Brien-Simpson N.M., Cecil J.D., Holden J.A., Reynolds E.C. (2014). Porphyromonas gingivalis outer membrane vesicles exclusively contain outer membrane and periplasmic proteins and carry a cargo enriched with virulence factors. J. Proteome Res..

[42] Fulsundar S., Domingues S., Nielsen K.M. (2019). Vesicle-Mediated Gene Transfer in Acinetobacter baumannii. Methods Mol. Biol..

[43] Kolling G.L., Matthews K.R. (1999). Export of virulence genes and Shiga toxin by membrane vesicles of Escherichia coli O157:H7. Appl. Environ. Microbiol..

[44] Perez-Cruz C., Delgado L., Lopez-Iglesias C., Mercade E. (2015). Outer-inner membrane vesicles naturally secreted by gram-negative pathogenic bacteria. PLoS ONE.

[45] Bitto N.J., Chapman R., Pidot S., Costin A., Lo C., Choi J., D’Cruze T., Reynolds E.C., Dashper S.G., Turnbull L. (2017). Bacterial membrane vesicles transport their DNA cargo into host cells. Sci. Rep..

[46] Chatterjee S., Mondal A., Mitra S., Basu S. (2017). Acinetobacter baumannii transfers the blaNDM-1 gene via outer membrane vesicles. J. Antimicrob. Chemother.

[47] Sjostrom A.E., Sandblad L., Uhlin B.E., Wai S.N. (2015). Membrane vesicle-mediated release of bacterial RNA. Sci. Rep..

[48] Koeppen K., Hampton T.H., Jarek M., Scharfe M., Gerber S.A., Mielcarz D.W., Demers E.G., Dolben E.L., Hammond J.H., Hogan D.A. (2016). A novel mechanism of host-pathogen interaction through sRNA in bacterial outer membrane Vesicles. PLoS Pathog..

[49] Furuse Y., Finethy R., Saka H.A., Xet-Mull A.M., Sisk D.M., Smith K.L., Lee S., Coers J., Valdivia R.H., Tobin D.M. (2014). Search for microRNAs expressed by intracellular bacterial pathogens in infected mammalian cells. PLoS ONE.

[50] Schwechheimer C., Sullivan C.J., Kuehn M.J. (2013). Envelope control of outer membrane vesicle production in Gram-negative bacteria. Biochemistry.

[51] Burdett I.D., Murray R.G. (1974). Electron microscope study of septum formation in Escherichia coli strains B and B-r during synchronous growth. J. Bacteriol..

[52] Eddy J.L., Gielda L.M., Caulfield A.J., Rangel S.M., Lathem W.W. (2014). Production of outer membrane vesicles by the plague pathogen Yersinia pestis. PLoS ONE.

[53] Roier S., Zingl F.G., Cakar F., Durakovic S., Kohl P., Eichmann T.O., Klug L., Gadermaier B., Weinzerl K., Prassl R. (2016). A novel mechanism for the biogenesis of outer membrane vesicles in Gram-negative bacteria. Nat. Commun..

[54] Gerritzen M.J.H., Maas R.H.W., van den Ijssel J., van Keulen L., Martens D.E., Wijffels R.H., Stork M. (2018). High dissolved oxygen tension triggers outer membrane vesicle formation by Neisseria meningitidis. Microb. Cell Fact.

[55] Cooke A.C., Nello A.V., Ernst R.K., Schertzer J.W. (2019). Analysis of Pseudomonas aeruginosa biofilm membrane vesicles supports multiple mechanisms of biogenesis. PLoS ONE.

[56] Florez C., Raab J.E., Cooke A.C., Schertzer J.W. (2017). Membrane Distribution of the Pseudomonas Quinolone Signal Modulates Outer Membrane Vesicle Production in Pseudomonas aeruginosa. mBio.

[57] Wessel A.K., Liew J., Kwon T., Marcotte E.M., Whiteley M. (2013). Role of Pseudomonas aeruginosa peptidoglycan-associated outer membrane proteins in vesicle formation. J. Bacteriol..

[58] Mashburn-Warren L., Howe J., Garidel P., Richter W., Steiniger F., Roessle M., Brandenburg K., Whiteley M. (2008). Interaction of quorum signals with outer membrane lipids: Insights into prokaryotic membrane vesicle formation. Mol. Microbiol..

[59] Li A., Schertzer J.W., Yong X. (2018). Molecular dynamics modeling of Pseudomonas aeruginosa outer membranes. Phys. Chem. Chem. Phys..

[60] Ellis T.N., Leiman S.A., Kuehn M.J. (2010). Naturally produced outer membrane vesicles from Pseudomonas aeruginosa elicit a potent innate immune response via combined sensing of both lipopolysaccharide and protein components. Infect. Immun..

[61] Beveridge T.J. (1999). Structures of gram-negative cell walls and their derived membrane vesicles. J. Bacteriol..

[62] Bauwens A., Kunsmann L., Marejkova M., Zhang W., Karch H., Bielaszewska M., Mellmann A. (2017). Intrahost milieu modulates production of outer membrane vesicles, vesicle-associated Shiga toxin 2a and cytotoxicity in Escherichia coli O157:H7 and O104:H4. Environ. Microbiol. Rep..

[63] Macdonald I.A., Kuehn M.J. (2013). Stress-induced outer membrane vesicle production by Pseudomonas aeruginosa. J. Bacteriol..

[64] Yanez-Mo M., Siljander P.R., Andreu Z., Zavec A.B., Borras F.E., Buzas E.I., Buzas K., Casal E., Cappello F., Carvalho J. (2015). Biological properties of extracellular vesicles and their physiological functions. J. Extracell. Vesicles.

[65] Pathirana R.D., Kaparakis-Liaskos M. (2016). Bacterial membrane vesicles: Biogenesis, immune regulation and pathogenesis. Cell Microbiol..

[66] Berleman J.E., Allen S., Danielewicz M.A., Remis J.P., Gorur A., Cunha J., Hadi M.Z., Zusman D.R., Northen T.R., Witkowska H.E. (2014). The lethal cargo of Myxococcus xanthus outer membrane vesicles. Front. Microbiol..

[67] Roier S., Zingl F.G., Cakar F., Schild S. (2016). Bacterial outer membrane vesicle biogenesis: A new mechanism and its implications. Microb. Cell.

[68] Cooke A.C., Florez C., Dunshee E.B., Lieber A.D., Terry M.L., Light C.J., Schertzer J.W. (2020). PQS-Induced outer membrane vesicles enhance biofilm dispersion in Pseudomonas aeruginosa. bioRxiv.

[69] Seike S., Kobayashi H., Ueda M., Takahashi E., Okamoto K., Yamanaka H. (2021). Outer membrane vesicles released from aeromonas strains are involved in the biofilm formation. Front. Microbiol..

[70] Toledo A., Coleman J.L., Kuhlow C.J., Crowley J.T., Benach J.L. (2012). The enolase of Borrelia burgdorferi is a plasminogen receptor released in outer membrane vesicles. Infect. Immun..

[71] Baarda B.I., Zielke R.A., Le Van A., Jerse A.E., Sikora A.E. (2019). Neisseria gonorrhoeae MlaA influences gonococcal virulence and membrane vesicle production. PLoS Pathog..

[72] Bielaszewska M., Ruter C., Bauwens A., Greune L., Jarosch K.A., Steil D., Zhang W., He X., Lloubes R., Fruth A. (2017). Host cell interactions of outer membrane vesicle-associated virulence factors of enterohemorrhagic Escherichia coli O157: Intracellular delivery, trafficking and mechanisms of cell injury. PLoS Pathog..

[73] Kuehn M.J., Kesty N.C. (2005). Bacterial outer membrane vesicles and the host-pathogen interaction. Genes Dev..

[74] Bielaszewska M., Marejkova M., Bauwens A., Kunsmann-Prokscha L., Mellmann A., Karch H. (2018). Enterohemorrhagic Escherichia coli O157 outer membrane vesicles induce interleukin 8 production in human intestinal epithelial cells by signaling via Toll-like receptors TLR4 and TLR5 and activation of the nuclear factor NF-kappaB. Int. J. Med. Microbiol..

[75] Cai W., Kesavan D.K., Wan J., Abdelaziz M.H., Su Z., Xu H. (2018). Bacterial outer membrane vesicles, a potential vaccine candidate in interactions with host cells based. Diagn. Pathol..

[76] Kaparakis-Liaskos M., Ferrero R.L. (2015). Immune modulation by bacterial outer membrane vesicles. Nat. Rev. Immunol..

[77] Rodrigues M., Fan J., Lyon C., Wan M., Hu Y. (2018). Role of Extracellular Vesicles in Viral and Bacterial Infections: Pathogenesis, Diagnostics, and Therapeutics. Theranostics.

[78] Domingues S., Nielsen K.M. (2017). Membrane vesicles and horizontal gene transfer in prokaryotes. Curr. Opin. Microbiol..

[79] Velimirov B., Ranftler C. (2018). Unexpected aspects in the dynamics of horizontal gene transfer of prokaryotes: The impact of outer membrane vesicles. Wien. Med. Wochenschr..

[80] Gaudin M., Krupovic M., Marguet E., Gauliard E., Cvirkaite-Krupovic V., Le Cam E., Oberto J., Forterre P. (2014). Extracellular membrane vesicles harbouring viral genomes. Environ. Microbiol..

[81] Medvedeva E.S., Baranova N.B., Mouzykantov A.A., Grigorieva T.Y., Davydova M.N., Trushin M.V., Chernova O.A., Chernov V.M. (2014). Adaptation of mycoplasmas to antimicrobial agents: Acholeplasma laidlawii extracellular vesicles mediate the export of ciprofloxacin and a mutant gene related to the antibiotic target. Sci. World J..

[82] Yaron S., Kolling G.L., Simon L., Matthews K.R. (2000). Vesicle-mediated transfer of virulence genes from Escherichia coli O157:H7 to other enteric bacteria. Appl. Environ. Microbiol..

[83] Fulsundar S., Harms K., Flaten G.E., Johnsen P.J., Chopade B.A., Nielsen K.M. (2014). Gene transfer potential of outer membrane vesicles of Acinetobacter baylyi and effects of stress on vesiculation. Appl. Env. Microbiol..

[84] Ho M.H., Chen C.H., Goodwin J.S., Wang B.Y., Xie H. (2015). Functional advantages of Porphyromonas gingivalis vesicles. PLoS ONE.

[85] Rumbo C., Fernandez-Moreira E., Merino M., Poza M., Mendez J.A., Soares N.C., Mosquera A., Chaves F., Bou G. (2011). Horizontal transfer of the OXA-24 carbapenemase gene via outer membrane vesicles: A new mechanism of dissemination of carbapenem resistance genes in Acinetobacter baumannii. Antimicrob. Agents Chemother..

[86] Blesa A., Berenguer J. (2015). Contribution of vesicle-protected extracellular DNA to horizontal gene transfer in Thermus spp. Int. Microbiol..

[87] Renelli M., Matias V., Lo R.Y., Beveridge T.J. (2004). DNA-containing membrane vesicles of Pseudomonas aeruginosa PAO1 and their genetic transformation potential. Microbiology.

[88] Tran F., Boedicker J.Q. (2017). Genetic cargo and bacterial species set the rate of vesicle-mediated horizontal gene transfer. Sci. Rep..

[89] Gill S., Katchpole R., Forterre P. (2019). Extracellular membrane vesicles in the three domains of life and beyond. FMES Microbiol. Rev..

[90] Tran F., Boedicker J.Q. (2019). Plasmid characteristics modulate the propensity of gene exchange in bacterial vesicles. J. Bacteriol..

[91] Marraffini L.A., Sontheimer E.J. (2008). CRISPR interference limits horizontal gene transfer in staphylococci by targeting DNA. Science.

